# Analytical Validation of an HPLC–DAD Method for Ivermectin Quantification in Polymeric Nanocapsules

**DOI:** 10.1021/acsomega.6c00602

**Published:** 2026-06-30

**Authors:** Mariana Alice Gonzaga Gabú, Mylena Lemos dos Santos, Laryssa Ferreira do Nascimento Silva, Paloma Manuelle Marques da Silva, Danilo César Galindo Bedor, Douglas Dourado, Fábio Rocha Formiga

**Affiliations:** † Department of Immunology, 92923 Aggeu Magalhães Institute (IAM), Oswaldo Cruz Foundation (FIOCRUZ), Recife, Pernambuco 50670-420, Brazil; ‡ Pharmaceutical and Cosmetic Development Center (NUDFAC), Department of Pharmaceutical Science, Federal University of Pernambuco, Recife, Pernambuco 50740-520, Brazil; § Faculty of Medical Sciences (FCM), University of Pernambuco (UPE), Recife, Pernambuco 50100-130, Brazil

## Abstract

Ivermectin (IVM) is a broad-spectrum antiparasitic drug whose clinical application is limited by poor aqueous solubility, chemical instability, and pharmacokinetic variability. Polymeric nanocapsules represent a promising strategy to overcome these limitations. However, their complex composition requires reliable and selective analytical methods for accurate drug quantification. In this study, a reversed-phase HPLC–DAD method was developed and validated for the quantification of ivermectin and applied to polymeric nanocapsules, including the determination of encapsulation efficiency and drug loading. Chromatographic separation was achieved on a C18 column using isocratic elution with acetonitrile and purified water (90:10, v/v) with diode-array detection at 253 nm and a retention time of approximately 4.7 min. The method was validated according to ICH Q2­(R2) guidelines, demonstrating adequate selectivity through evaluation of matrix interference and analysis under stress conditions. Linearity was observed over the analytical range (R^2^ > 0.99), while accuracy and precision were satisfactory, with recovery values close to 100% and relative standard deviation below 2%. Application of the method to polymeric nanocapsules resulted in high encapsulation efficiency. A preliminary evaluation of chemical stability over 30 days indicated that ivermectin content remained above 95% under the evaluated storage conditions. Overall, the proposed method is simple, selective, precise, accurate, robust, and suitable for the quantitative analysis of ivermectin in complex nanostructured formulations.

## Graphical abstract

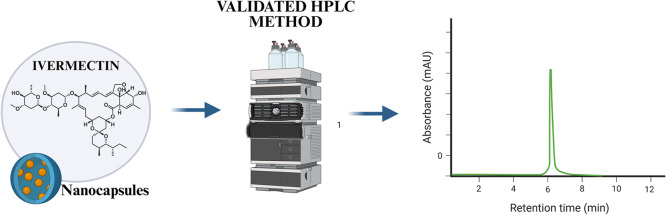

## 1 Introduction

Ivermectin (IVM) is a macrocyclic lactone derived from avermectin (Figure 1)^1,2^ and has been widely used in human and veterinary antiparasitic therapies due to its broad-spectrum activity against helminths and ectoparasites.^1,3^ In addition, IVM has demonstrated antiviral activity against certain DNA and RNA viruses.^4^

**Figure 1:**
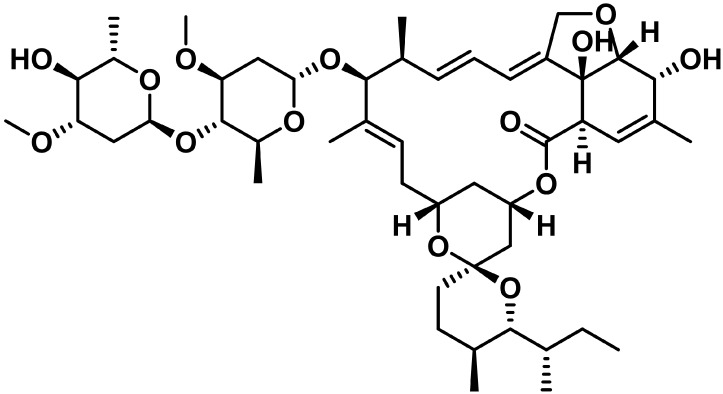
Chemical structure of ivermectin. The structure was drawn using ChemDraw.

Despite its biological properties, IVM presents physicochemical and biopharmaceutical drawbacks. It is classified as a Biopharmaceutical Classification System (BCS) class II drug, characterized by low aqueous solubility and high intestinal permeability.^5^ Furthermore, ivermectin exhibits chemical instability and high affinity for plasma proteins, which may contribute to pharmacokinetic variability, reduced maximum plasma concentration (*C*_max_), and inconsistent therapeutic efficacy. Repeated or prolonged administration of ivermectin has also been associated with decreased therapeutic efficacy and an increased incidence of adverse events, according to pharmacological and clinical evidence. This underscores the need for pharmaceutical strategies that can improve drug delivery while minimizing systemic toxicity.^6,7^

To overcome these limitations, nanotechnology systems have been employed.^8^ Among them, polymeric nanocapsules are composed of an oily core, surfactants, and a polymer coating. These systems are capable of increasing the solubility and permeability of active pharmaceutical ingredients (APIs), protecting against degradation and modulating the release profile, reducing systemic toxicity, and increasing the therapeutic efficacy of drugs such as ivermectin.^9–12^ However, the complex compositions of such nanostructured systems, including polymer, surfactants and oily presents significant analytical challenges, requiring robust and selective methods capable of accurately quantifying the drug in complex matrices.

In this context, High-Performance Liquid Chromatography (HPLC) is widely used in the quantification of pharmaceuticals compounds due to its high sensitivity, specificity, and reproducibility.^13,14^ However, for the reliability of analytical results, it depends on proper method development and validation. Although specific regulatory guidelines for drug quantification in nanostructured systems are still limited, method validation is typically performed based on established frameworks for conventional pharmaceutical products, such as ANVISA Resolution RDC N° 166/2017, ICH Q2­(R2) and ICH Q14,^15,16^ which establishes key evaluation parameters, including selectivity, linearity, accuracy, precision, and limits of detection and quantification.

Thus, a validated and matrix-specific analytical method is essential to ensure reliable drug quantification in ivermectin-loaded polymeric nanocapsules. Therefore, this study aimed to develop and validate an HPLC–DAD method for the quantitative determination of ivermectin in poly­(ε-caprolactone)-based nanocapsules and to apply it to the determination of encapsulation efficiency. In addition, a preliminary assessment of the chemical stability of ivermectin in the nanocapsule formulation was performed to further demonstrate the applicability of the method. This approach provides analytical support for nanotechnology-based pharmaceutical development and quality control applications.

## 2 Materials and Methods

### 2.1 Chemicals and Reagents

Ivermectin (purity: 93% B1a ivermectin and 3% B1b ivermectin), poly­(ε-caprolactone) (PCL, Mw = 80,000), sorbitan monooleate (Span 80), and polysorbate 80 (Tween 80) were purchased from Sigma-Aldrich (St. Louis, MO, USA). Pumpkin seed oil was obtained from *Mundo dos Óleos* (São Paulo, SP, Brazil). HPLC-grade acetonitrile (GOLD Ultragradient grade) was supplied by Carlo Erba Reagents (Val de Reuil, France). Ultrapurified water was obtained from a water purification system (Milli-Q Direct 8, Merck Millipore, Darmstadt, Germany). Hydrochloric acid (HCl), sodium hydroxide (NaOH), and hydrogen peroxide (H_2_O_2_), used in the evaluation of selectivity under stress conditions, were of analytical grade and obtained from standard commercial suppliers. All other reagents were of analytical grade and used as received.

### 2.2 Instrumentation and Chromatography Conditions

The High-Performance Liquid Chromatography (HPLC) system consisted of a Shimadzu LC-2050C 3D model equipped with a Diode Array Detector (DAD). Chromatographic separation was performed using a Zorbax Eclipse C18 analytical column (4.6 mm × 150 mm, 5 μm particle size). The mobile phase consisted of acetonitrile and purified water (90:10, v/v), delivered at a flow rate of 1.5 mL/min under isocratic elution. The injection volume was 20 μL, and the column temperature was maintained at 25 °C. Detection was performed at 243 nm, a wavelength selected based on the absorption spectrum of IVM. The analyses were carried out over 6.5 min. Data acquisition and processing were performed using the LabSolutions software (Shimadzu, Japan).

### 2.3 Ivermectin Standards Solutions

The ivermectin (IVM) stock solutions were prepared by dissolving 10 mg of IVM in acetonitrile in a 10 mL volumetric flask, completing the volume to obtain a final concentration of 1000 μg/mL. The solutions were filtered through a 0.22 μm polytetrafluoroethylene (PTFE) syringe filter.

### 2.4 Synthesis of Polymeric Nanocapsules

Nanocapsules (NCs) were prepared using the nanoprecipitation method^17,18^ (Figure 2). Briefly, the organic phase, composed of 87.5 mg of pumpkin seed oil, 20 mg of PCL, 12 mg of Span 80, and 5 mg of ivermectin, was solubilized in 6 mL of acetone. Then, this organic phase was slowly injected, drop by drop, into 7 mL of the aqueous phase (Milli-Q water) containing 12 mg of Tween 20, under magnetic stirring for 20 min at 50*g* and 25 °C (Fisher-Bioblock Scientific AM 3001 K, Illkirch, France). The resulting dispersion was concentrated using a rotary evaporator for 40 min (3*g*, 40 °C, 65 mbar) (BÜCHI Rotavapor R-125, Heating Bath B-491, Vacuum Pump V-700, Cooler F-108, Flawil, Switzerland) to remove the solvent. To obtain the empty nanocapsules, the same process was carried out, without the addition of the drug. Finally, the nanocapsules were stored at 4 °C.

**Figure 2:**
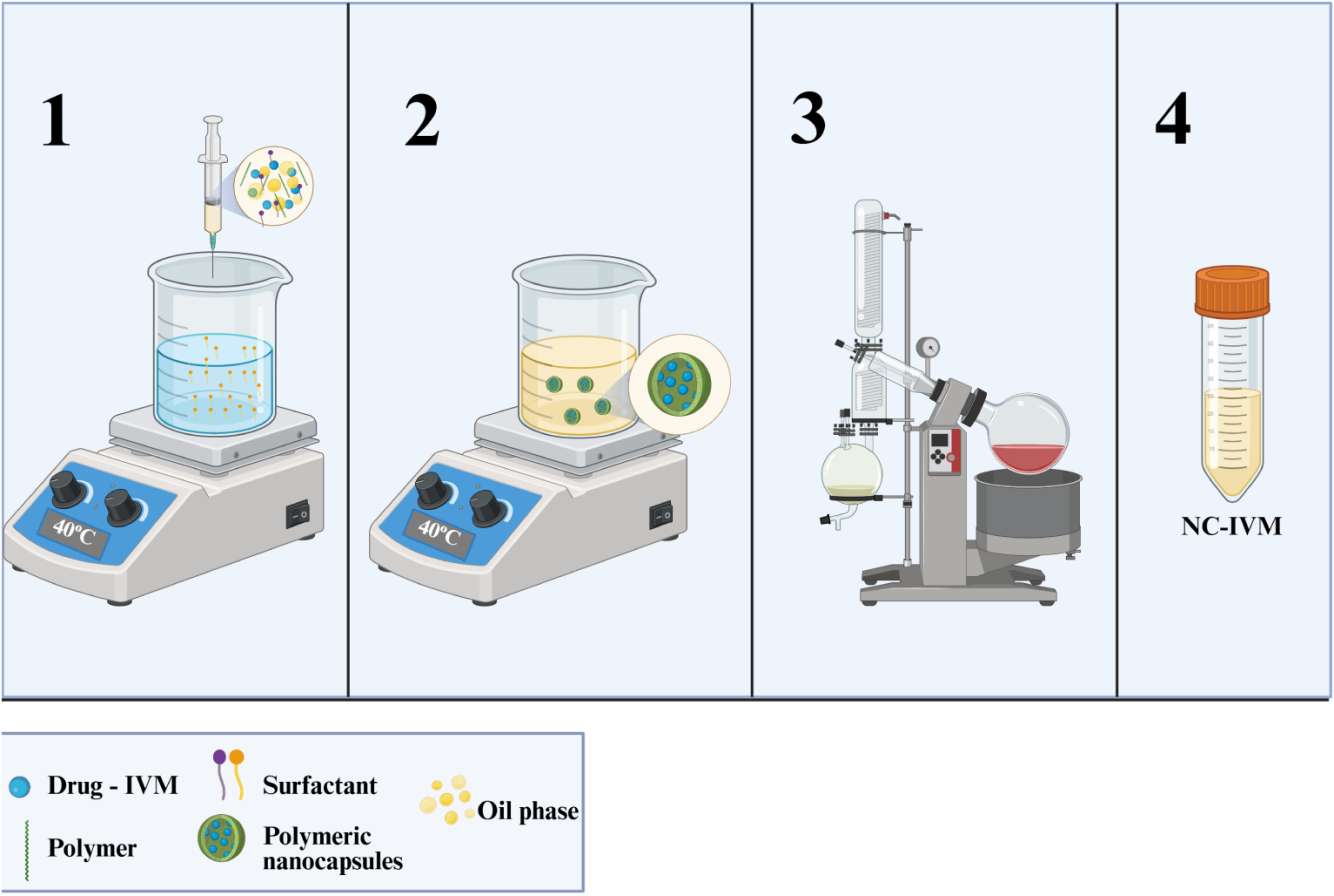
Nanocapsules preparation by nanoprecipitation. Created in https://BioRender.com (2026).

### 2.5 Physicochemical Characterization of the Nanoparticles

The hydrodynamic particle size, polydispersity index (PdI), and zeta potential (ZP) of the nanoparticles were evaluated at 25 °C, using a fixed light scattering angle of 90°, with a Zetasizer Nano ZS (Malvern Instruments, United Kingdom). Before measurement, all samples were diluted in purified water at a 1:100 (v/v) ratio.

### 2.6 System Suitability

Before the determination of encapsulation efficiency (EE), system suitability was evaluated to verify adequate chromatographic system performance. The assessment was carried out by consecutive injections of a standard solution of ivermectin under the optimized chromatographic conditions. The parameters evaluated included peak area, retention time (tR), asymmetry factor (As), number of theoretical plates (N), and capacity factor (k′). The relative standard deviation (RSD%) was calculated for each parameter in order to assess the system repeatability. The system was considered suitable when the obtained values demonstrated acceptable repeatability and chromatographic performance, in accordance with international recommendations.

### 2.7 Method Validation

Method validation was conducted in accordance with the International Council for Harmonisation (ICH) guideline Q2­(R2), for analytical method validation.^16^ The method was evaluated in terms of selectivity, linearity, limit of detection (LOD), limit of quantification (LOQ), precision (repeatability and intermediate precision), accuracy, and robustness. Before each validation assay, system suitability criteria were assessed to ensure adequate chromatographic performance, including evaluation of parameters such as (I) number of theoretical plates (N), (II) tailing factor, (III) resolution, and repeatability of peak area, in accordance with ICH Q2­(R2) recommendations.^16^ Each validation parameter was systematically assessed according to ICH Q2­(R2) criteria, and the corresponding experimental results, acceptance criteria, and statistical analyses are presented in the following sections to demonstrate that the method provides reliable, accurate, and precise quantification of ivermectin.

#### 2.7.1 Selectivity

The selectivity of the method was evaluated against matrix components and under stress conditions.

##### 2.7.1.1 Selectivity against Matrix Components

To evaluate the selectivity of the method in the presence of the nanoparticle matrix, ivermectin-loaded nanocapsules (IVM-NCs) and empty nanocapsules (Empty-NCs) were prepared. Both formulations were subjected to the same nanoparticle disruption process using acetonitrile, followed by filtration through a 0.22 μm PTFE syringe filter. The resulting samples were analyzed by HPLC to assess potential matrix interferences and to verify whether the chromatographic conditions allowed for the selective detection of ivermectin without coelution of the formulation components.

##### 2.7.1.2 Selectivity under Stress Conditions

IVM solutions (500 μg/mL) were subjected (1:1 v/v) to acidic (0.1 M HCl, 24 h), oxidative (3% v/v H_2_O_2_, 24 h), and alkaline (0.1 M NaOH, 6 h). Under alkaline conditions, extensive degradation was observed after prolonged exposure; therefore, a shorter exposure time (6 h) was selected to allow adequate evaluation of method selectivity. Additionally, IVM solutions (250 μg/mL) were exposed to thermal stress (60 °C, oven) and photolytic stress (direct exposure to an 18 W LED lamp) for 24 h. After the exposure periods, the samples were filtered through a 0.22 μm (PTFE) syringe filter and analyzed by HPLC. These experiments were performed to generate potential degradation products and to verify whether the IVM peak remained free from interference under stress conditions, thereby supporting the assessment of method selectivity.

#### 2.7.2 Linearity, Limit of Detection (LOD), and Limit of Quantification (LOQ)

The linearity of the method was evaluated through the correlation between concentration and analytical response. To this end, three independent calibration curves were prepared with IVM standard solutions, using a concentration range from 31.25 μg/mL to 1000 μg/mL. Linearity was analyzed using the correlation coefficient (R) and the coefficient of determination (R^2^), based on the average values from the three calibration curves. Additionally, the limit of detection (LOD) and the limit of quantification (LOQ) were determined using the eqs 1 and 2 below:

1 $$\mathrm{LOD}=\frac{3.3\times \sigma }{S}$$

2 $$\mathrm{LOQ}=\frac{10\times \sigma }{S}$$

Wherein σ is the estimated standard deviation, and *S* is the slope of the analytical curve.

#### 2.7.3 Precision

Precision was evaluated in terms of repeatability (intraday) and intermediate precision (interday). Repeatability was assessed using six determinations at the 100% test concentration. Intermediate precision was performed by analyzing solutions prepared by 2 different analysts, each performing the analysis on two different days. For comparison between days, Student’s *t* test was applied. In all evaluations, precision was expressed as the relative standard deviation (RSD%).

#### 2.7.4 Accuracy

Accuracy was evaluated by recovery studies at three concentration levels corresponding to 80%, 100%, and 120% of the nominal test concentration, classified as low (200 μg/mL), medium (250 μg/mL), and high (300 μg/mL), respectively. For each level, three independent replicates were prepared, totaling nine determinations (n = 9). Recovery was calculated using the equation below:

3 $$\%=\frac{\mathrm{recovered concentration}}{\mathrm{theoretical added concentration}}\times 100$$

#### 2.7.5 Robustness

Robustness was evaluated by introducing small, deliberate variations in critical chromatographic parameters, including mobile phase flow rate (±0.1 mL/min), proportion of organic solvent (acetonitrile) in the mobile phase (±1%), and column temperature (±2 °C). For each modified condition, chromatographic parameters were analyzed: retention time (tR), peak area, tailing factor, capacity factor (k′), and separation factor were evaluated. The results were assessed based on predefined system suitability criteria.

### 2.8 Method Application

#### 2.8.1 Encapsulation Efficiency (EE%) and Drug Loading (DL%)

Encapsulation efficiency was determined using the direct and indirect method.^19,20^

To quantify the total drug fraction (FT), 1 mL of the nanoparticle dispersion was mixed with acetonitrile, followed by centrifugation and filtration through a 0.22 μm PTFE syringe filter. The resulting filtrate was then analyzed by HPLC.

For the direct method, 1 mL of the nanoparticle’s suspension, was subjected to vacuum concentrator at 40 °C (2000*g*) to remove the aqueous phase and concentrated the nanoparticles fraction. The resulting dried pellet (FD) was resuspended in acetonitrile to disrupt the nanocapsules and extract the associated drug, followed by centrifugation, filtration (0.22 μm membrane) and subsequent HPLC analysis. Additionally, to verify the presence of nonencapsulated drug, an indirect method based on ultrafiltration (Vivaspin, 100 kDa, at 4500*g* for 45 min) was also performed. Briefly, the nanoparticle suspension was placed in centrifugal ultrafiltration devices and centrifuged (40 min 4 °C 4500*g*) to separate the free drug fraction from the nanoparticle-associated fraction. The filtrate was collected and analyzed by HPLC to quantify the nonencapsulated ivermectin (FI).

Encapsulation efficiency (EE%) was calculated according to eqs 4 and 5. The experiments were performed using five independent nanoparticles batches prepared under identical conditions to assess the reproducibility of the formulation process and the reliability of the analytical methods.:

4 $$\mathrm{EE}\;\left(\%\right)=\frac{\mathrm{FT}-\mathrm{FD}}{\mathrm{FT}}\times 100$$

5 $$\mathrm{EE}\;\left(\%\right)=\frac{\mathrm{FT}-\mathrm{FI}}{\mathrm{FT}}\times 100$$

Drug loading (DL%) was calculated based on the amount of encapsulated drug relative to the total mass of the nanocapsule formulation, as described in eq 6:

6 $$\mathrm{DL}\;\left(\%\right)=\frac{\mathrm{mass}\;\mathrm{of}\;\mathrm{drug}\;\mathrm{encapsulated}}{\mathrm{total}\;\mathrm{mass}\;\mathrm{of}\;\mathrm{nanoparticles}}\times 100$$

#### 2.8.2 Preliminary Stability Study of Ivermectin-Loaded Nanocapsules

A preliminary stability study was conducted to evaluate the chemical stability of ivermectin in polymeric nanocapsules during storage. Nanocapsule suspensions containing ivermectin at a concentration of 500 μg/mL were stored under two temperature conditions (4 ± 2 °C and 25 ± 2 °C) for 30 days.

Samples were collected at predetermined time intervals (0, 7, 15, and 30 days) and analyzed using the validated HPLC method. For analysis, aliquots of the nanocapsules suspension were diluted 1:1 (v/v) with acetonitrile, resulting in a final concentration of 250 μg/mL, corresponding to the 100% level of the calibration curve. Under these conditions, the nanocapsules were disrupted following the same procedure described for the encapsulation efficiency determination, allowing the extraction of ivermectin and the quantification of the total drug content.

The samples were centrifuged and filtered through a 0.22 μm membrane prior to HPLC analysis. The experiments were performed using five independent nanocapsules batches (n = 5) prepared under the same conditions. The chemical stability of the ivermectin in the formulation was expressed as the percentage of the initial ivermectin content, considering the concentration at day 0 as 100%.

## 3 Results and Discussion

### 3.1 Physicochemical Characterization of the Polymeric Nanocapsules (NCs)

Empty-nanocapsules (Empty-NCs) and IVM-loaded nanocapsules (IVM-NCs) presented average particle sizes of 384.24 ± 3.8 nm and 410.60 ± 10.97 nm, respectively (Table 1). The slight increase observed in the size of the NC-PCL-IVM suggests the incorporation of the drug into the polymeric core, without compromising the homogeneity of the system.

**Table 1:** Physicochemical Characterization of Nanocapsules (NCs)^a^

	Size (nm)	PdI	Zeta potential (mV)
Empty-NCs	384.24 ± 3.80	0.10 ± 0.08	–36.4 ± 0.98
IVM-NCs	410.60 ± 10.97	0.09 ± 0.05	–33.2 ± 0.27

a Source: prepared by the authors (2025).

Additionally, both formulations presented polydispersity index (PdI) values lower than 0.3, indicating a monodisperse particle distribution, as also evidenced by the histograms presented in Figure 3. These results demonstrate the good reproducibility of the preparation process and are compatible with physicochemically stable colloidal systems, in which low PdI values are directly associated with the population uniformity of the nanoparticles.^21^

**Figure 3:**
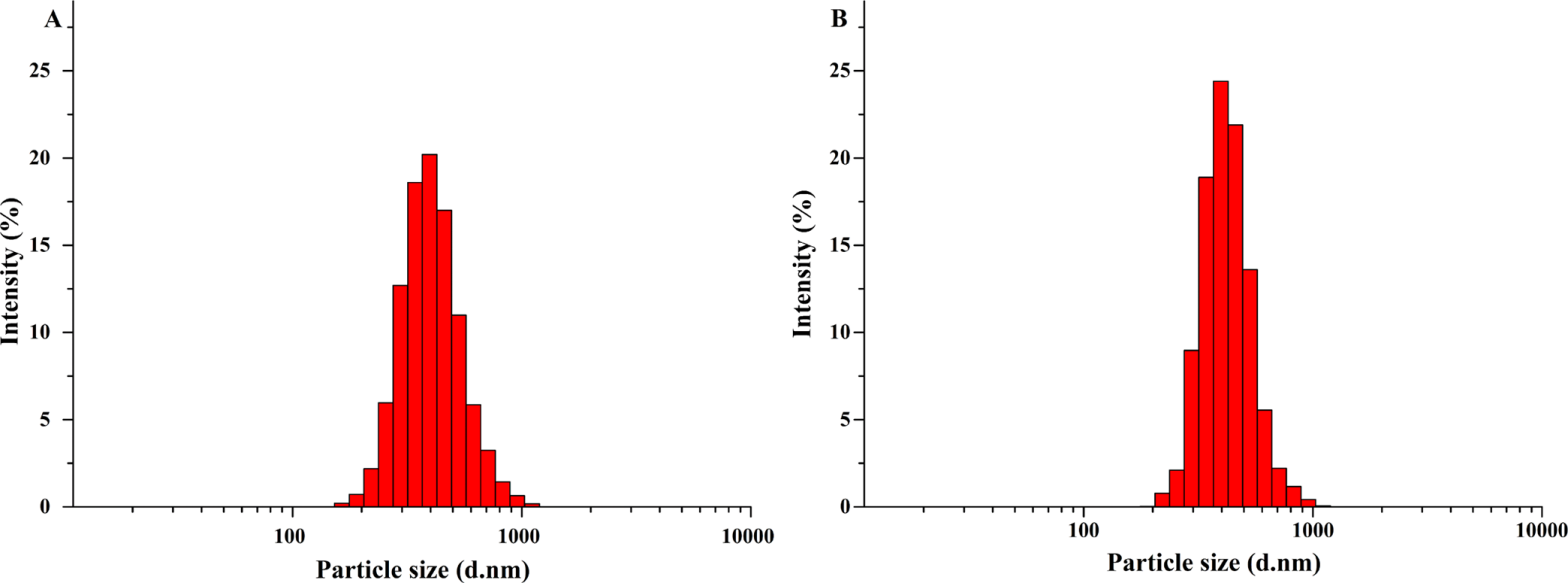
Size distribution of nanocapsules (NCs). (A) Empty-nanocapsules (Empty-NC); (B) ivermectin-loaded nanocapsules (IVM-NC).

Regarding the zeta potential, both formulations showed negative values, −36.4 ± 0.98 mV for Empty-NCs and −33.2 ± 0.27 mV for IVM-NCs. The absence of a significant difference between the zeta potential values indicates that the incorporation of ivermectin did not substantially alter the surface charge of the nanoparticles, suggesting that the drug is predominantly encapsulated within the polymeric matrix, without relevant exposure on the surface. The observed negative zeta potential is associated with the anionic nature of the PCL polymer surface, as well as the presence of the surfactant polysorbate 80, which contributes to both the electrostatic and steric stabilization of the system, due to its bulky and highly hydrophilic structure.^12,22^

The physicochemical properties of the developed nanoparticles directly influence their interaction with biological barriers and, consequently, their potential therapeutic performance. According to Xu et al. (2022), the size of the nanoparticles is a critical determinant for oral absorption, since systems with diameters smaller than 500 nm have a greater capacity to cross the intestinal mucus layer, be internalized by enterocytes, and reach the portal circulation, where they are subject to first-pass hepatic metabolism.^23^

Furthermore, the negative surface charge observed for the nanocapsules (approximately −33 mV) may contribute to reduced recognition by cells of the mononuclear phagocytic system, favoring a longer systemic circulation time and, potentially, better bioavailability of the encapsulated drug.^24,25^

In summary, the particle size, PdI, and zeta potential results confirm the achievement of homogeneous, stable nanocapsules suitable for ivermectin incorporation, providing a solid basis for analytical and performance evaluations of the formulation.

### 3.2 Chromatographic Conditions and System Suitability

Analytical methods applied to nanocarrier systems must be developed and validated to ensure analytical quality. Although matrix interference is a common concern in the analysis of different pharmaceutical dosage forms, it becomes even more critical in nanoformulations due to their complex composition and the potential interactions between the drug, polymers, and other excipients. Therefore, particularly in chromatographic techniques such as HPLC, validation must be performed using the representative matrix of the final formulation.^26^

Therefore, the selection of chromatographic conditions was conducted aiming to maximize selectivity, reduce analysis time, and ensure reproducibility, as recommended for the analysis of complex pharmaceutical matrices.^27^

The C18 column was selected due to its recognized suitability for the analysis of hydrophobic compounds, such as ivermectin, providing appropriate retention and peak symmetry.^28^

On the other hand, acetonitrile was chosen as an organic modifier because it presents high reproducibility, lower system pressure, and good ivermectin solubilization capacity, in addition to favoring the resolution of the formulation constituents.^29^

After a preliminary evaluation of solvent proportions and flow rate, an isocratic elution system using acetonitrile and water (90:10, v/v) was chosen to facilitate routine analyses and reduce the total run time. The final operating conditions were a flow rate of 1.5 mL min^–1^ and a column temperature of 25 °C, which resulted in stable retention times and adequate performance of the chromatographic system.^18,27^

Finally, the suitability of the chromatographic system was evaluated using system suitability parameters, including retention time, asymmetry factor, retention factor, resolution, number of theoretical plates, signal-to-noise ratio, and peak purity. The results obtained (Table 2) demonstrate that the established chromatographic conditions and the system performance are adequate for the quantification of ivermectin, meeting the system suitability requirements for the validation of the analytical method.

**Table 2:** Preliminary Parameters of the Chromatographic System

Analytical parameters	Result
Retention time (Rt)	4.869 min
Asymmetry factor (As)	0.830
Retention factor (k′)	3.961
Resolution (Rs)	1.336
Number of theoretical plates (N)	1794
Signal-to-noise ratio (S/N)	6958.89
Peak purity index	0.995213

### 3.3 Method Validation

#### 3.3.1 Selectivity

The selectivity of the method was evaluated by comparing the chromatograms obtained for the ivermectin standard solution (IVM), in absence and under stress conditions, as well as for polymeric nanocapsules containing ivermectin (IVM-NCs) and empty nanocapsules (Empty-NCs). The chromatograms are presented in Figure 4.

**Figure 4:**
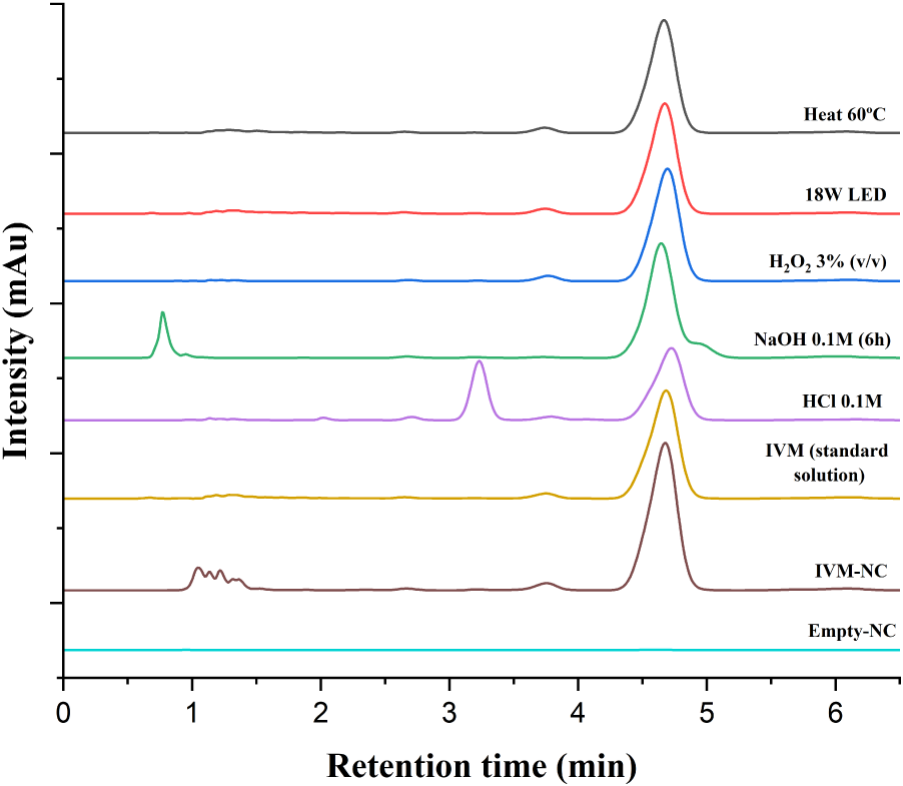
Selectivity evaluation of the HPLC–DAD method for ivermectin.

The standard ivermectin solution showed a well-defined peak, with a mean retention time of approximately 4.7 min, a value consistent with those reported in the literature, in which IVM typically elutes in the time interval between 4 and 10 min.^30,31^ The variations observed between the different methods are related to differences in the chromatographic conditions employed, including column dimensions, particle size, composition, concentration, and number of mobile phase solvents, flow rate, temperature, and elution mode.^32^

It is important to highlight that, while previously described methods employ more complex chromatographic systems such as the use of three solvents, acidified mobile phases, or gradient elution, the method proposed in this study uses a simple isocratic system, composed of only two solvents (acetonitrile and purified water), providing lower operational cost, greater analytical simplicity, and reduced retention time.

Under stress conditions, a reduction in the peak area corresponding to ivermectin was observed in acidic and alkaline media, along with the emergence of additional peaks at different retention times. In an acidic medium, the formation of a product eluting at a shorter retention time is consistent with the cleavage of the saccharide residue of the molecule, a mechanism widely described for ivermectin.^33^ In an alkaline medium, more pronounced degradation was observed, with the appearance of multiple peaks eluting early, a behavior similar to that reported in the literature for basic conditions.^34^ On the other hand, no alterations were observed in the chromatographic profile of IVM under oxidative, photolytic, and thermal stress conditions, corroborating studies that report greater stability of IVM under these conditions when dissolved in solvents such as acetonitrile.^23^ It is important to highlight that no coelution was observed during the ivermectin retention time. This was corroborated by peak purity analysis using diode array detection (DAD), which confirmed that the analyte peak was spectrally homogeneous and free of coeluting components, demonstrating that the method is capable of selectively quantifying the analyte in the presence of potential degradation products.

Additionally, the chromatogram obtained for the empty nanocapsules (Empty-NCs) did not show peaks eluting at the same retention time as ivermectin, demonstrating the absence of interference from the formulation excipients. Similarly, the ivermectin extracted from the IVM-NCs showed a characteristic peak, without coelution with matrix components.

Collectively, these results demonstrate that the method is selective for the detection and quantification of ivermectin in the presence of formulation components and potential degradation products generated under stress conditions. These findings are consistent with ICH Q2­(R2) recommendations for selectivity assessment in assay methods.

#### 3.3.2 Linearity, Limit of Detection (LOD), and Limit of Quantification (LOQ)

The linearity of the method was evaluated by constructing three independent analytical curves using six ivermectin concentration levels. The curves showed a significant linear relationship between analyte concentration and peak area, as demonstrated by the high coefficient of determination (R^2^ > 0.999) obtained for the average analytical curve.

Linear regression was statistically evaluated by analysis of variance (ANOVA), which confirmed that the slope coefficient is significantly different from zero (*p* < 0.0001), evidencing a statistically significant linear correlation between the analyzed variables. The limits of detection (LOD) and quantification (LOQ) were determined based on the standard deviation of the response and the slope of the calibration curve, in accordance with ICH Q2­(R2) recommendations. The linear regression parameters, as well as the limit of detection (LOD) and limit of quantification (LOQ) values, are presented in Table 3. Thus, these results demonstrate that the method presents adequate linearity, sensitivity, and statistical reliability for the quantification of ivermectin under the evaluated conditions, according to the criteria established ICH Q2­(R2) guidelines.^16^

**Table 3:** Linear Regression Equation, Coefficient of Determination (R^2^), LOD, and LOQ of the Analytical Method

Linear regression equation	y = 22357x + 221033
R^2^	0.9993
LOD	15.25 μg/mL
LOQ	50.84 μg/mL

#### 3.3.3 Precision

The evaluation of method precision, expressed as relative standard deviation (RSD%), demonstrated satisfactory results (Table 4). For repeatability (intraday precision), the mean RSD% values obtained by each analyst individually were below 2%, indicating good consistency among measurements performed on the same day. Similarly, intermediate precision (interday precision) also showed mean RSD% values below 2%. The low RSD (%) values obtained for both repeatability and intermediate precision demonstrate the consistency and reliability of the analytical method. These results comply with the criteria established by ICH Q2­(R2) guidelines, which recommend acceptable RSD (%) values to ensure the precision of quantitative analytical methods, and are consistent with findings reported in the literature.^35^

**Table 4:** Repeatability and Intermediate Precision of the Analytical Method

Experimental condition	n	Mean (%)	SD (%)	RSD (%)
Repeatability (Intraday)	6	98.05	±0.60	0.61
Intermediate precision (Interday/Analyst 1)	12	98.27	±0.61	0.62
Day 1	6	98.10	±0.59	0.62
Day 2	6	98.50	±0.58	0.59
Intermediate precision (Interday/Analyst 2)	12	99.92	±1.13	1.13
Day 1	6	99.69	±0.50	0.50
Day 2	6	99.90	1.56	1.56

#### 3.3.4 Accuracy

The accuracy of the method was evaluated through recovery studies at three concentration levels (80%, 100%, and 120% of the nominal concentration), as shown in Table 5.

**Table 5:** Determined Values for Accuracy Analysis

Peak level (%)	Theoretical concentration (μg/mL)	Mean area	Standard deviation	Coefficient of variation (%)	Determined concentration (μg/mL)	Recovery (%)
80	200	4640186.67	7755.60	0.167	197.66	98.83
100	250	5780313.00	5880.58	0.102	248.66	99.46
120	300	6855704.67	7025.07	0.102	296.76	98.92

Average recovery values ranged from 98.83% to 99.46%, demonstrating good agreement between the theoretically and experimentally determined concentrations. Furthermore, the results showed low variability, with relative standard deviation (RSD%) values below 2% at all levels. These results demonstrate the accuracy of the method for the quantification of ivermectin across the evaluated concentration range. These findings are consistent with the acceptance criteria reported in the literature and recommended by ICH Q2­(R2), confirming that the method provides adequate accuracy for the quantitative analysis of ivermectin.^16,36^

#### 3.3.5 Robustness

The robustness of the method was evaluated through small, deliberate variations in chromatographic conditions, including column temperature (23 and 27 °C), mobile phase flow rate (1.3 and 1.7 mL min^–1^), and acetonitrile (ACN) proportion in the mobile phase (89% and 91%). The monitored parameters were retention time, peak area, asymmetry factor, and resolution, as recommended by ICH Q2­(R2) guidelines. The results obtained (Table 6) reveal that the variations evaluated did not promote significant changes in chromatographic performance.

**Table 6:** Robustness Evaluation of the HPLC–DAD Method for Ivermectin Quantification^a^

Condition	Retention time (min) Mean ± SD (CV%)	Peak area Mean (CV%)	Tailing factor Mean (CV%)	Resolution (Rs) Mean (CV%)
Normal	4.871 ± 0.011 (0.22)	5.24 × 10^6^ (0.68)	0.831 (0.17)	1.336 (0.11)
Temp. 23 °C	5.048 ± 0.045 (0.90)	5.18 × 10^6^ (1.27)	0.848 (2.29)	1.337 (0.65)
Temp. 27 °C	4.631 ± 0.043 (0.92)	5.18 × 10^6^ (1.34)	0.855 (2.23)	1.308 (1.77)
Flow 1.3 mL min^–1^	5.571 ± 0.007 (0.13)	6.02 × 10^6^ (0.59)	0.814 (0.21)	1.311 (2.35)
Flow 1.7 mL min^–1^	4.267 ± 0.026 (0.60)	4.58 × 10^6^ (0.75)	0.860 (1.55)	1.325 (1.70)
ACN 89%	5.061 ± 0.006 (0.11)	5.13 × 10^6^ (0.03)	0.835 (0.98)	1.341 (0.04)
ACN 91%	4.551 ± 0.007 (0.16)	5.14 × 10^6^ (0.01)	0.870 (1.95)	1.283 (1.43)

a Values expressed as mean ± SD (n = 3). CV: coefficient of variation.

The coefficients of variation remained below 2% for all critical parameters, indicating adequate repeatability and stability of the analytical system in the face of the studied variations.^15,36^ The ivermectin peak remained well-defined and symmetrical, with asymmetrical factor values within acceptable limits, as well as sufficient resolution to ensure reliable quantification of the analyte. These results demonstrate the robustness of the method, as small variations in chromatographic conditions did not significantly affect its analytical performance. Thus, the method exhibits adequate robustness, being able to withstand small operational variations without compromising its analytical performance, which makes it suitable for application in the quantification of ivermectin.

### 3.4 Comparison with Reported HPLC Methods

Several HPLC methods for the determination of ivermectin have been reported in the literature, differing mainly in terms of sensitivity, analysis time, and chromatographic complexity (Table 7). The method developed in this study is also included in Table 7 to enable direct comparison with previously reported approaches. Overall, the method developed in this study stands out for its simplicity and rapid analysis while maintaining adequate performance for routine quantitative applications.

**Table 7:** HPLC Methods Comparation

Method (Ref.)	Matrix	Mobile phase	Elution mode	Retention time (min)	Column	Detection conditions	LOD/LOQ	Main limitation
Heredero et al., 2023 ^37^	Feed, soil, water	Acetonitrile:methanol:water (56:37:7, v/v/v)	Isocratic	8.1	C18 Supelcosil (250 × 4.6 mm, 5 μm)	245 nm; 100 μL; 20 °C; 1.2 mL/min	3.2–12.5 μg/kg; 9–30 μg/kg	Ternary mobile phase
Kumar et al., 2023 ^38^	Pharmaceutical dosage form	Methanol:phosphate buffer pH 3 (70:30, v/v)	Isocratic	2.34	ACE C18 (150 × 4.6 mm, 5 μm)	240 nm; 1.2 mL/min	3.17 μg/mL; 5.68 μg/mL	pH control
Padivitage et al., 2023 ^28^	Oral paste	Water (A)/acetonitrile:methanol (85:15, v/v) (B)	Gradient	–	Zorbax Extend-C18 (150 × 4.6 mm, 3.5 μm)	245 nm; 30 °C; 1.5 mL/min	0.2 μg/mL; 0.6 μg/mL	Gradient elution
Wimalasinghe et al., 2021 ^29^	IVM	Water–acetonitrile (50:50, v/v) (A); isopropanol–acetonitrile (15:85, v/v) (B)	Gradient	–	Ascentis Express C18 (100 × 4.6 mm, 2.7 μm)	252 nm; 45 °C	0.3 μg/mL; 1.0 μg/mL	Gradient elution
**This work**	IVM and IVM-loaded polymeric nanocapsules	Acetonitrile:water (90:10, v/v)	Isocratic	∼4.7	Zorbax Eclipse-C18 (150 × 4.6 mm, 5 μm)	243 nm; 25 °C; 1.5 mL/min	15.25 μg/mL; 50.84 μg/mL	Lower sensitivity compared to trace-level methods

Compared to the method proposed by Heredero et al. 2023, which employs a ternary mobile phase, large injection volume, and an internal standard, the present method adopts a simpler approach, using a binary mobile phase, lower injection volume, and shorter run time. Although the use of an internal standard improves analytical precision, the simplification proposed here enhances practicality and reduces solvent and sample consumption.^37^

The method described by Kumar et al. 2023 shows good sensitivity and a comparable run time; however, it requires pH control and involves preparation of the mobile, which may increase variability. In contrast, in the present study, the mobile phase was prepared manually prior to analysis, allowing better control of composition and improved reproducibility. Additionally, the developed method operates under milder and more robust conditions for routine use, albeit with lower sensitivity.^38^

More complex approaches, such as those reported by Padivitage et al. 2023 and Wimalasinghe et al. 2021, rely on gradient elution and are designed for advanced applications, including impurity profiling and stability studies. These methods provide higher selectivity but at the expense of longer run times and increased operational complexity. In contrast, the proposed isocratic method, with a retention time of approximately 4.7 min and a total run time of 6.5 min, is more suitable for high-throughput routine analysis, although it is not intended for separation of degradation products or isomers.^28,29^

In addition, official compendial methods described in pharmacopoeias such as the United States Pharmacopeia and the European Pharmacopoeia are widely adopted in the pharmaceutical industry due to their regulatory acceptance across multiple countries. However, these methods are generally based on conventional chromatographic conditions, typically employing 25 cm long columns packed with fully porous particles of 5 μm. Such configurations are often associated with longer retention times and limited chromatographic efficiency and selectivity, which can reduce their suitability for routine quality control environments. In contrast, more recent strategies in method development have focused on improving efficiency and reducing analysis time through the use of modern stationary phases and optimized chromatographic conditions. In this context, the method developed in this study aligns with the need for more QC-friendly approaches by offering a faster and simpler alternative without compromising its applicability for routine quantitative analysis.^39,40^

In summary, the proposed method offers key advantages in terms of speed, simplicity, and ease of implementation, making it well-suited for routine applications. Although it presents lower sensitivity and selectivity compared to some literature and pharmacopoeial methods, it proved to be suitable for the intended purpose and was successfully applied to the quantification of ivermectin in polymeric nanocapsules.

### 3.5 Application of the Method

#### 3.5.1 Encapsulation Efficiency (EE%) and Drug Loading (DL%)

The analytical method developed and validated for ivermectin (IVM) quantification was applied to determine the drug content in a polymeric nanocapsule formulation. After evaluation of all relevant validation parameters, the method demonstrated adequate applicability for this purpose, as illustrated in Figure 5, enabling the reliable determination of encapsulation efficiency and drug loading.

**Figure 5:**
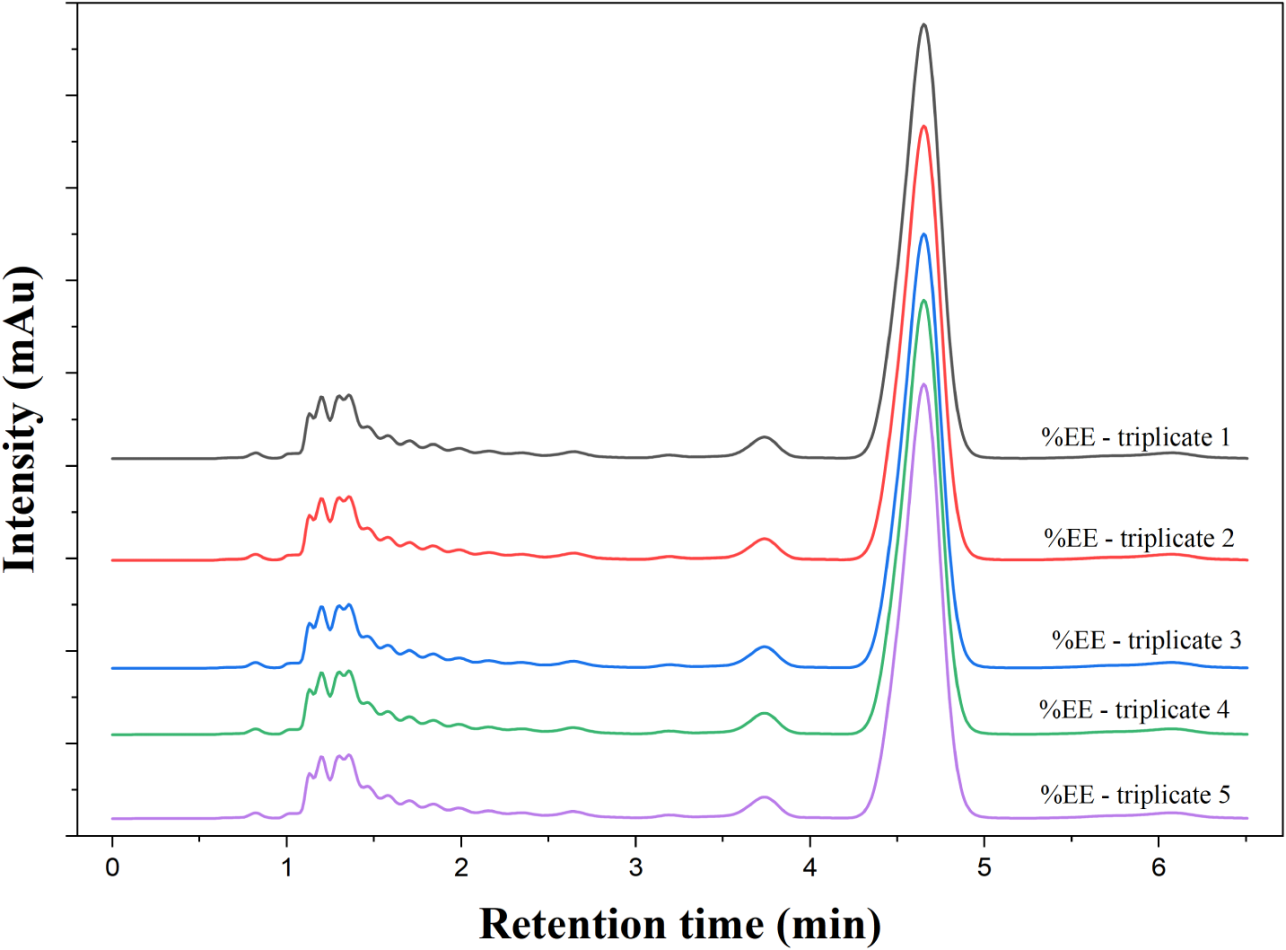
Chromatographic profiles show the measurement of encapsulation efficiency (%EE) for ivermectin-loaded nanocapsules (IVM-NC) by direct method.

The developed formulation exhibited a high encapsulation efficiency (EE%) of 94.83 ± 1.54% (RSD = 1.62%, n = 5), indicating that most of the ivermectin was efficiently incorporated into the polymeric nanocapsules. The low relative standard deviation observed among independent batches demonstrates good reproducibility of the formulation process and adequate precision of the analytical method. These results are consistent with international analytical validation guidelines, which recommend RSD values below 2% for quantitative chromatographic methods, confirming the reliability of the proposed method and the robustness of the encapsulation process.^15^ It is important to note that the direct approach involves a solvent removal step prior to drug extraction, which does not rely on the volatility of ivermectin, but rather on the concentration of the nanoparticle fraction. In this context, although the method enables effective quantification of the drug associated with the nanocapsules, a potential contribution of nonencapsulated drug to the recovered fraction cannot be completely excluded from a theoretical standpoint.

To address this limitation, an indirect method based on ultrafiltration was employed to evaluate the presence of free drug. Ivermectin levels in the filtrate were below the limit of quantification of the validated method, with no detectable peaks observed under the established chromatographic conditions. These findings indicate that the nonencapsulated fraction is negligible and that any potential interference of free drug in the direct method is minimal under the experimental conditions used. Thus, the direct and indirect methods consistently support a high encapsulation efficiency of IVM in nanoparticles.

High EE% values are generally associated with favorable interactions between the drug and the polymeric matrix components, which contribute to formulation stability and favor the protection and controlled release of the active pharmaceutical ingredient (API).^41,42^ Recent studies further demonstrate that strong drug–polymer affinity and optimized formulation parameters play a key role in achieving high encapsulation efficiency, physicochemical stability, and controlled release behavior in polymeric nanocarrier systems.^21,43^

The robustness of the encapsulation results is further supported by the use of a direct quantification method, which involves disruption of the nanocapsule’s structure followed by drug extraction and chromatographic analysis. Direct methods have been shown to provide a more accurate estimation of the actual drug payload within nanocarriers compared to indirect approaches, particularly for hydrophobic drugs prone to adsorption or incomplete separation of the free fraction.^19^

Encapsulation efficiencies comparable to those obtained in this study have been reported for polymeric nanoparticle systems containing ivermectin. Mohammed et al. reported an EE% of 93.99 ± 0.96%, whereas other studies described lower efficiencies, around 53%, depending on formulation composition and preparation method.^5,44^ Therefore, the EE% achieved in the present work demonstrates superior or at least comparable performance relative to previously reported ivermectin-loaded polymeric nanocarriers.

Based on the encapsulation efficiency obtained, the drug loading (DL%) was calculated, resulting in a value of approximately 4%. This DL% is consistent with values typically reported for polymeric nanocapsule systems designed for hydrophobic drugs. Although higher drug loading values are often desirable, moderate DL% values are commonly associated with improved physicochemical stability, reduced drug leakage, and enhanced control over drug release profiles in polymeric nanocarrier systems. In this context, the DL% observed in the present study reflects a balanced formulation strategy, prioritizing high encapsulation efficiency and system stability rather than excessive drug loading.

It is also noteworthy that, in terms of drug concentration, the developed formulation contains ivermectin at levels above those reported as pharmacologically active in experimental models, reinforcing its potential applicability.^45,46^ Similar drug loading values (3–5%) associated with high encapsulation efficiencies and stable formulations have been reported for ivermectin-loaded polymeric nanoparticles. Taken together, these results support the suitability of the developed polymeric nanocapsule system for ivermectin administration.

#### 3.5.2 Preliminary Stability Study of Ivermectin-Loaded Nanocapsules

Solution stability is essential to ensure reliable and reproducible analytical results, particularly in situations involving extended analytical runs or the need for sample reanalysis. Maintaining sample integrity over time, under appropriate storage conditions, is therefore critical.^47^ In this context, the stability of ivermectin-loaded nanocapsules was assessed over a 30-day period by monitoring the percentage of ivermectin content (Table 8).

**Table 8:** Preliminary Stability of Ivermectin-Loaded Nanocapsules for 30 Days^a^

	% (Ivermectin content) (n = 5)		RSD (%) (n = 5)
Day	25 °C ± 2 °C	4 °C ± 2 °C	(25 °C|4 °C)
D0	99.90	99.98	0.13|0.2
D7	99.64	99.86	0.46|0.53
D15	99.94	97.98	1.25|1.32
D30	97.84	99.54	2.03|1.98

a There were no significant differences compared to D0 (*p*> 0.05).

The preliminary chemical stability study of ivermectin-loaded nanocapsules demonstrated high retention of drug content over the 30-day period under both storage conditions (25 ± 2 °C and 4 ± 2 °C), indicating good chemical stability of ivermectin within the nanostructured system. Drug content remained close to 100% during the first 15 days, suggesting minimal degradation and adequate short-term stability.

From D15 onward, a slight decrease in ivermectin content was observed under both storage conditions. However, no statistically significant differences were found compared to D0 (*p* > 0.05).

Nanocapsules (NCs) are versatile systems capable of enhancing the photo- and chemical stability of compounds. These findings are consistent with the literature, as reported by de Souza et al. (2023), who observed that ivermectin-loaded nanocapsules maintained stable drug content for up to 180 days without significant changes.^18,48^

Importantly, the relative standard deviation (RSD%) values remained below 2% throughout the study, indicating low variability, good analytical precision, and homogeneity of the samples consistent with the precision criteria established according to ICHQ2­(R2) during method validation.^16,35^

## 4 Conclusion

The validated HPLC–DAD method demonstrated selectivity, accuracy, precision and robustness for the reliable quantification of ivermectin in polymeric nanocapsules, confirming its suitability for application in complex nanotechnology systems. The method proved appropriate for determining drug content and encapsulation efficiency, contributing to quality control and ensuring the reliability of pilot scale formulations during pharmaceutical development.

From a scientific and regulatory perspective, this study provides a reproducible analytical tool, developed and validated according to ICH Q2­(R2), capable of selectively quantifying ivermectin in the presence of potential transformation products, as demonstrated under stress conditions. Additionally, the method shows potential for application in other stages of nanostructured system development, such as *in vitro* release studies, provided that appropriate evaluation or partial revalidation is performed, considering the specific characteristics of different simulated biological media.

Therefore, the proposed method can serve as a reference for future studies requiring validated analytical methods for quantitative determination in nanostructured drug delivery systems, contributing to the advancement of robust analytical practices in pharmaceutical nanotechnology.
